# Association between Picky Eating Behaviors and Nutritional Status in Early Childhood: Performance of a Picky Eating Behavior Questionnaire

**DOI:** 10.3390/nu9050463

**Published:** 2017-05-06

**Authors:** Kyung Min Kwon, Jae Eun Shim, Minji Kang, Hee-Young Paik

**Affiliations:** 1 Department of Food and Nutrition, College of Human Ecology, Seoul National University, 1 Gwanak-ro, Gwanak-gu, Seoul 08826, Korea; ckyme@snu.ac.kr (K.M.K.); hypaik@snu.ac.kr (H.-Y.P.); 2 Department of Food and Nutrition, Daejeon University, 62 Daehak-ro, Dong-gu, Daejeon 34520, Korea; 3 Daejeon Dong-gu Center for Children’s Food Service Management, Daejeon University, 62 Daehak-ro, Dong-gu, Daejeon 34520, Korea; 4 Research Institute of Human Ecology, College of Human Ecology, Seoul National University, 1 Gwanak-ro, Gwanak-gu, Seoul 08826, Korea; mjkang@snu.ac.kr

**Keywords:** picky eating, early childhood, diet, growth

## Abstract

Picky eating behaviors are frequently observed in childhood, leading to concern that an unbalanced and inadequate diet will result in unfavorable growth outcomes. However, the association between picky eating behaviors and nutritional status has not been investigated in detail. This study was conducted to assess eating behaviors and growth of children aged 1–5 years from the Seoul Metropolitan area. Primary caregivers completed self-administered questionnaires and 3-day diet records. Differences in the nutrient intake and growth indices between picky and non-picky eaters were tested by analysis of covariance. Children “eating small amounts” consumed less energy and micronutrients (with the exception of calcium intake), but picky behaviors related to a “limited variety” resulted in a significant difference regarding nutrient density for some micronutrients. Children with the behavior of “eating small amounts” had a lower weight-for-age than that of non-picky eaters; especially, the older children with the behaviors of “eating small amounts” or “refusal of specific food groups” had lower height-for-age compared with non-picky eaters. These results suggest that specific picky eating behaviors are related to different nutrient intake and unfavorable growth patterns in early childhood. Thus, exploration of potential interventions according to specific aspects of picky eating and their efficacy is required.

## 1. Introduction

Picky eating is a frequent eating problem in childhood that concerns many parents [1,2,3,4]. In young children, picky eating can contribute to a poor dietary intake and growth status [2,4,5,6] and may have long-term effects [7,8,9,10]. A recent review presented conflicting reports on dietary intake patterns in picky eating children: some studies reported an increased intake of energy or energy-dense foods including snacks and sweets, while most studies reported a limited variety of food intake with reduced energy consumption [11]. Both patterns could cause inappropriate changes in the nutrient composition of the diet and are related to unfavorable growth (i.e., poor growth and overweight) and subsequent health problems [11,12,13,14].

However, previous approaches to evaluating picky eaters are insufficient to explain the conflicting reports on dietary intake patterns, and investigate the association with growth outcomes. In previous studies, caregivers of picky-eating children reported various problems with feeding them: eating insufficient amounts, avoiding new foods, preferring foods prepared in specific ways, or having a strong preference for particular foods [2,4,6,8,15,16,17,18]. Picky eating is a complex concept composed of several types of eating behaviors [19]; nevertheless, picky eating has generally been measured by a single simple question based on parents’ perceptions of feeding difficulty or pickiness [2,18], or by a list of questions about eating behaviors and feeding practices [4,7,16,20,21]. The differences in measurements regarding picky eating focusing on one aspect or approaches using measurement tools consisting of mixed concepts leads to confusion and problems in interpretation [22].

Two recent studies have tried to present a clear definition of picky eating, and have characterized children’s picky eating behaviors with two attributes based on previously-reported aspects of picky eating behaviors: eating small amounts of food, and eating a limited variety of foods [19,23]. In the studies, “limited variety” consisted of three sub-constructs of “unwillingness to try new food”, “rejection of specific food groups” (i.e., fruits, vegetables, meats, and fish), and “preference for specific food preparation methods”. To find critical behaviors in child growth, the association of the four aspects of picky behaviors and growth in young children was examined at a medical clinic for picky eaters [23]. The medical clinic study measured the level of the four aspects of picky eating behaviors using similar questions to the present study (i.e., the same questions for the measurement of “eating small amounts” and “neophobic behavior”, 9 vs. 12 food groups for “refusal of specific food groups”, and 7 vs. 9 food groups for preference for specific food preparation method). In the study, negative association between “eating small amounts” and height-for-age was observed. Difference in nutrient intake and the relations with growth outcomes in a community setting have not yet been examined.

Thus, the present study investigated the picky eating behaviors of the four constructs in children aged 1 to 5 years at the community level. Further, the performance using the four-construct scale was evaluated qualitatively by examining how the four different aspects of picky eating were associated with dietary intake and growth. It is hypothesized that each aspect would have a specific pattern in dietary intake and growth.

## 2. Materials and Methods

### 2.1. Study Participants

This study is a cross-sectional survey targeting children aged 1 to 5 years from the Seoul Metropolitan Area of Korea. Participants were recruited between September 2014 and July 2015. Convenience sampling was employed to recruit volunteers by using flyers, public announcements, and online announcements at Community Health centers, a pharmacy, and an online caregiver’s community. Voluntarily participating primary caregivers of the children were asked to complete the survey questionnaire. Participants were enrolled after the caregivers were given a full explanation of the purpose and protocols of the research in person. The Seoul National University Institutional Review Board approved the study protocol (IRB No. 1407/001-034), and all primary caregivers provided written informed consent.

### 2.2. Measurements

#### 2.2.1. Picky Eating Behaviors

Picky eating behaviors were assessed using survey questions from previous studies [19,23]. Caregivers were asked to respond to the frequency of each question using a five-point response scale of 1 (almost never) to 5 (almost always). The higher scores demonstrated greater picky eating behavior, so the reverse-described questions were transposed. Self-administered surveys were reviewed by a trained dietitian and confirmed by interview. The four specific picky eating behaviors and the related questions were:

- Eating small amounts, with the question of “How often do you attempt to persuade your child to eat a food?”, and two reversed-described questions: “In general, at the end of a meal how often has your child eaten the amount you think he/she should eat?” and “Does your child have a good appetite?”
- Neophobic behavior, with two reverse-described questions: “How often does your child try new and unfamiliar foods at home?”, and “How willing is your child to enjoy new and unfamiliar food when offered?”
- Refusal to eat specific food groups, using the question on 12 food groups: “How often does your child refuse the following foods: beans, vegetables, mushrooms, seaweeds, meat, fish, shrimp, shellfish, eggs, fruits, milk, and yogurt?”
- Preference for a specific food preparation method, with the question on nine food groups: “Does your child eat any of the following foods only if prepared in a specific way: beans, vegetables, mushrooms, seaweeds, meat, fish, shrimp, shellfish, and eggs?”

It was assumed that the children have potential picky eating characters if the response score to each question was higher than neutral. “Eating small amounts” and “neophobic behavior” were summated rating scales. Therefore, the children whose mean score of responses was >3 were classified as “picky eaters” for “eating small amounts” and “neophobic behavior”. The internal consistency of items on these constructs was measured using the Cronbach’s coefficient α (α = 0.80 for “eating small amounts” and α = 0.73 for “neophobic behavior”). Whether children refused a food group or whether children had preference for a specific preparation method to a food group was also determined by a response score > 3. However, “refusal to eat specific food groups” and “preference for a specific food preparation method” were not summated rating scales. The constructs consisted of multiple questions about behaviors to different food groups. Therefore, “refusal to eat specific food groups” and “preference for a specific food preparation method” were evaluated based on the number of foods refused and number of foods with specific preparation method preferred, respectively. The cut-off number was set based on the mean numbers of food groups with responses more than neutral (1.8 for refused food groups and 1.2 for preference for a specific food preparation method). Therefore, children who refused more than two food groups were classified as picky eaters of “refusal to eat specific food groups” and children with a preference for a specific food preparation method in any food group were categorized as picky eaters of “preference for a specific food preparation method”. If certain food groups had never been tried, the food groups were not counted when “refusal to eat specific food groups” or “preference for a specific food preparation method” was evaluated. Children who had any one of the sub-constructs, “neophobic behavior”, “refusal to eat specific food groups”, and “preference for a specific food preparation method” were defined as children with ‘limited variety’. Children who had any one of the picky eating main constructs, “eating small amounts” and “limited variety”, were classified as picky eaters.

#### 2.2.2. Dietary Intakes

Non-consecutive 3-day diet records were used to collect the dietary intake data of each subject. To minimize errors in portion size, the caregivers were asked to record the intake amount by using two-dimensional measurement tools. The protocol for coding diet records was prepared by a research dietitian supervisor. Based on the protocol, trained dietitian interviewers reviewed the data by telephone interview. For children who were still being breastfed, the intake of breastmilk was assessed according to the reported feeding time; the amount being fed was considered to be 1 fl. oz. (29.6 mL) for every 5 min [24]. All dietary data were converted to nutrient intake values using the DES-KOREA (Diet Evaluation System, 2011, Human Nutrition Lab at Seoul National University, Republic of Korea), which is a web-based dietary assessment program [25,26]. The DES incorporates a recipe and nutrient database. The recipe database contains 3916 recipes for common Korean dishes, and the nutrient database contains 4222 food items [27,28]. The mean daily intake, the energy distribution for macronutrients, the nutrient density (intake/1000 kcal of energy) for micronutrients, and the total dietary fiber were evaluated.

#### 2.2.3. Growth Indices

Primary caregivers were asked to measure the weight and height of their children at the local hospital or community health center and report the values to the research dietitian by mail. Confirmation was made through telephone interviews. The height data for children aged ≤24 months were converted into lengths by adding 0.7 cm, following the WHO (World Health Organization) child growth standards [29]. The height/length and weight values were converted into *z*-scores for weight-for-age, height-for-age (length-for-age), and BMI (body mass index)-for-age, compared with the WHO child growth standards for 0–60 months [29] and the WHO growth reference data for 61–228 months [30].

#### 2.2.4. Covariates

A questionnaire investigating the children’s eating behaviors, feeding practices, and care environment was administered, and data were obtained by self-reporting via the caregivers of the participating children. The sociodemographic characteristics included information pertaining to the caregivers, such as age, education level of both parents (≤high school, college graduate, graduate school), and monthly household income (≤$2800, $2800 to $3900, and ≥$3900), and information pertaining to the children, such as age and sex. In addition, Nutrition Plus participation (a nutrition supplemental program for women, infants and children in Korea) and infant feeding practices were investigated. Infant feeding practice was evaluated according to the duration of breastfeeding, the introduction of formula or milk, and the introduction of supplementary foods. This information was transformed into binary variables, including breastfeeding initiation, exclusive breastfeeding during the first 3 months and 6 months of life, and early introduction of supplementary foods before 6 months of age [31].

### 2.3. Statistical Analysis

The data of sociodemographic characteristics and the prevalence of picky eating habits were presented as numbers and proportions for categorical variables or as means and standard deviations for numeric variables. The differences in nutrient intake and *z*-scores of growth indices between picky eaters and non-picky eaters in each construct were tested by analysis of covariance to adjust for the child’s age and sex and the education level of both parents, after examination of covariates as potential confounders [32]. All the statistical analyses were performed using SAS (version 9.3, 2011, SAS Institute Inc., Cary, NC, USA), and the statistical significance was determined at 0.05.

## 3. Results

### 3.1. Sociodemographic Characteristics of Study Participants

Among the 221 children of the caregivers who initially volunteered and were eligible for the study, 14 with missing data from their diet records and 1 who had consecutive food records were excluded. An additional 22 participants were excluded because of food restrictions due to food allergies, a vegetarian diet, or religious beliefs, leaving 184 children with complete data.

As shown in Table 1, participants generally lived in well-educated middle-class families. Approximately 39% of children participated in Nutrition Plus. The growth indices of all participants were within the normal ranges.

### 3.2. Proportion of Picky Eaters

The proportion of participants with the behavior of “eating small amounts” was 29.9% and with the “limited variety” was 66.9%; with the “preference for a specific food-preparation method” was 49.5%, with the “refusal to eat specific food groups” was 44.0%, with the “neophobic behavior” was 32.6% (Table 1). In addition, compared with the younger children, the older children aged 4 to 5 years showed higher rates of eating behaviors related to a variety of foods, especially “neophobic behavior” (47.5% vs. 25.6%, *p* = 0.0032). Most children showed more than one kind of picky behavior: of the children with the behavior of “eating small amounts”, 67.3% also displayed a “refusal to eat specific food groups” and 43.6% “neophobic behavior”; of the children with “neophobic behavior”, 40.0% exhibited “eating small amounts” and 75.0% a “refusal to eat specific food groups”; of the children with a “refusal to eat specific food groups”, 45.7% exhibited “eating small amounts” and 55.6% “neophobic behavior”; of the children with “preference for a specific food-preparation method”, 63.7% exhibited a “refusal to eat specific food groups”; 9.8% of the children exhibited all of these picky eating behaviors, while 29.9% had none of the picky eating behaviors (data not shown).

**Figure 1:**
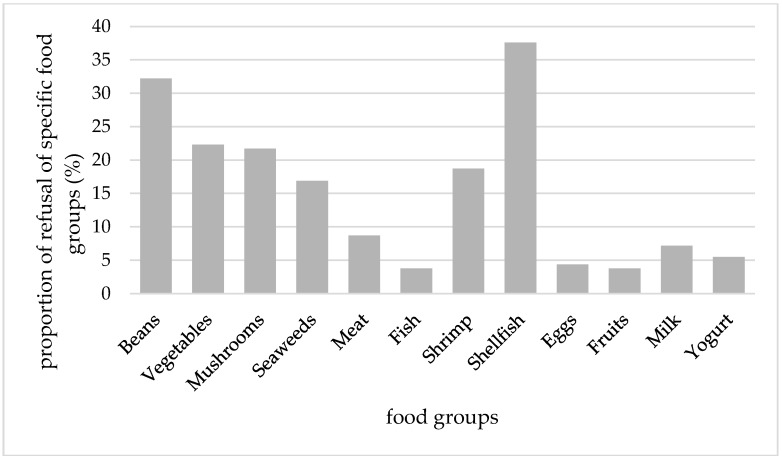
The proportion of children who usually refused a specific food group. The descriptive statistics for the distribution of number of food groups refused as follows: mean ± SD = 1.8 ± 1.9, Q_1_ = 0, median = 1, and Q_3_ = 3.

**Figure 2:**
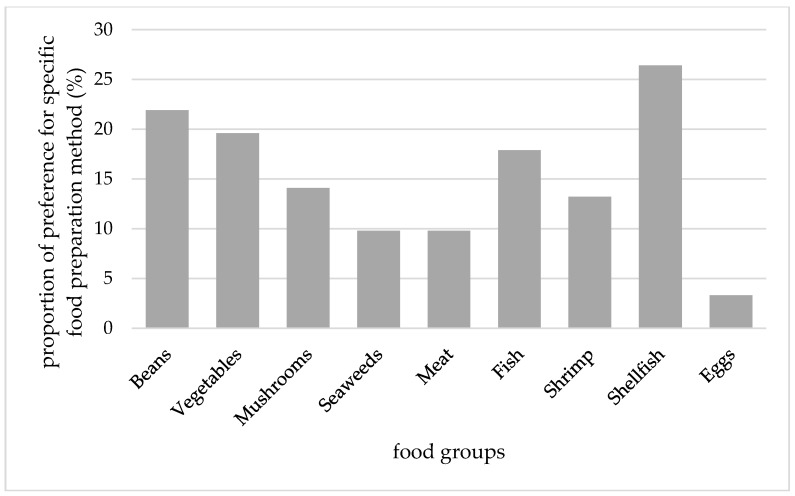
The proportion of children who usually requested food preparation in a certain way for each food group. The descriptive statistics for the distribution of number of food groups with specific preparation as follows: mean ± SD = 1.2 ± 1.8, Q_1_ = 0, median = 0, and Q_3_ = 1.

The proportions of children who refused each food groups and who preferred specific preparation for each food groups are shown in Figure 1 and Figure 2. The three most frequently refused food groups were shellfish, beans, and vegetables, and the three least refused food groups were fish, fruits, and eggs. Children required foods to be prepared in a certain way—mainly for shellfish and beans. Only 3% of children required eggs to be prepared in a certain way. Fish was not likely to be refused; however, it was required to be prepared in a certain way.

### 3.3. Comparison of the Dietary Intake and Growth Indices between Picky Eaters and Non-Picky Eaters

#### 3.3.1. Dietary Intake

The characteristics of the dietary intake of picky eaters—as compared to non-picky eaters—varied with each eating behavior (Table 2). Children considered to be “eating small amounts” had a significantly lower intake of energy and all micronutrients, with the exception of calcium intake. With respect to the picky eating behavior of a limited variety, there was no significant difference in energy intake between picky and non-picky eaters. The children with “neophobic behavior” consumed less dietary fiber per 1000 kcal of energy intake than did their counterparts. Picky eaters with a “refusal of specific food groups” consumed less micronutrients, with the exception of calcium and niacin intake. There was also a significant difference in nutrient density with some micronutrients. The “preference for a specific food preparation method” was related to lower intakes of iron and vitamin A.

#### 3.3.2. Growth Indices

The comparison of growth indices between picky eaters and non-picky eaters are presented in Table 3. Picky eaters “eating small amounts” had lower *z*-scores for weight-for-age (*p* = 0.0010) and BMI-for-age (*p* = 0.0278) but lower scores for height-for-age, with marginal significance (*p* = 0.054). Picky eaters “eating small amounts” aged 4 to 5 years had significantly lower *z*-scores for all three growth indices. Picky eaters with “refusal of specific food groups” were related with lower height-for-age in this age group.

## 4. Discussion

Research on picky eating faces difficulties due to a lack of widely-accepted definitions and appropriate measurement tools. Different definitions used by different researchers may indicate that picky eating behavior is not simple, but rather has complex characteristics that cannot be defined by one single aspect. Thus, in the present study, different aspects of picky eating were clarified and then measured and evaluated separately for their associations with nutritional status. The results suggested that picky eating behavior consists of different constructs showing specific nutrient intake and growth patterns, and the measurement tool could be used to investigate picky eating behaviors and the associated outcomes.

The present study adopted the previous approach to measure the two main constructs of “eating small amounts” and “limited variety” in picky eating behaviors, and the three sub-constructs of “neophobic behavior”, “refusal of specific food groups”, and “preference for a specific preparation method” in “limited variety” [19,23]. “Eating small amounts” refers to consuming insufficient food, and “limited variety” includes “neophobic behavior”, which refers to avoiding new foods and a “refusal of specific food groups” as well as a “preference for a specific preparation method”, which refers to children’s likes and dislikes of specific foods and certain recipes for each food [19,23]. While children show more than one construct behavior simultaneously and the classification of children overlapped, this approach could find a specific association between children’s eating behaviors and diet and growth.

Children “eating small amounts” consumed less energy and nutrients and had lower scores for growth indices compared with non-picky eaters in the present study. The frequently reported behaviors of picky eating children were spitting food out, eating avoidance, or throwing food, which may lead to “eating small amounts” [33]. Additionally, caregivers who experienced feeding difficulties reported that their children had a low appetite [34]. These fussy behaviors—which lead to consuming less food—were related to dietary problems in the present study. In previous studies, children classified as picky eaters had lower intakes of energy and nutrients, such as vitamin E, folate, and dietary fiber [2,4,5,17], and the children had slower growth rates and gained less weight [2,6,12]. However, the previous studies did not try to identify which specific picky eating behaviors were associated with the nutrient intakes and the growth outcomes.

A longitudinal study reported that children who are picky eaters are more likely to have a low BMI-for-age [12]. The risk was likely to increase when the picky eating problem continued as the children became older [30]. In the present study, it was observed that association between growth indices and picky eating behaviors was more prominent in the older children than in the younger children. Moreover, in the present study, the children with picky eating habits in the older age group had shorter heights and lower BMIs than those in the younger age group, indicating the need for further examination whether unfavorable long-term growth outcomes would be induced by picky eating behaviors.

In other studies, food neophobia and food rejection were related with limited preference for all food groups—especially vegetables and fruits [35,36]. In the present study, picky eaters with behaviors related to choosing a limited variety of foods had a lower quality of diet for some micronutrients, but not energy. However, the “refusal of some food groups” was related to lower height-for-age among children aged 4 to 5 years in this study. This suggests the necessity for further investigation on long-term problems induced by food avoidance, in terms of the negative influence of micronutrient deficiency on linear growth. Younger picky eaters with “neophobic behavior” were likely to have a lower *z*-score for height-for-age (*p* = 0.0657) and a higher *z*-score for BMI-for-age (*p* = 0.0575). If food neophobia is not appropriately countered at the period of introduction of complementary food, some food groups may remain refused throughout the life. Thus, in younger children, food neophobia and the long-term impact on growth may be concerns, even though energy consumption is not compromised.

It has been reported that the main reason for rejecting foods is distaste and dislike of color [37,38]. Cooking changes the color, taste, and texture of foods. Many of the children with “preference for a specific food preparation” had the picky eating behavior of “refusal of some food groups”, while their dietary intake and growth indices were not compromised. The most dramatic change of children’s food choice was fish. It indicated that if children could find an appropriate preparation for disliked foods then they might choose to eat the foods with the preparation. These findings imply that an appropriate food preparation method that positively influences food intake would be helpful for the prevention of poor growth. The impact of “preference for a specific food preparation method” needs to be assessed in further studies.

However, there are some limitations to this study. It was conducted as a cross-sectional study of a well-educated, small-scale sample living in a metropolitan area. Thus, this study was not free from counter causality (i.e., smaller children with less appetite behaving in a way to be classified as picky eaters), and generalizability of the results is limited. Potential confounders were not fully evaluated and controlled for; a few socio-demographic characteristics were controlled for, but other potential covariates such as the child’s characteristics and child feeding practices were not. The study variables were measured by the caregiver’s report, which represented personal values and expectations. Additionally, separate evaluation of “refusal to eat specific food groups” and “preference for a specific food preparation method” was a novel approach in this area. Therefore, evidence for relevant question forms and response cut-off criteria was scarce. Mean numbers of food groups were adopted as cut-off values, but might seem to be arbitrary. Currently, the two sub-constructs were not absolutely diagnosed, but relatively; nevertheless, association with growth status was observed. Especially strength of the association was dependent on the duration of picky eating behaviors. Further examinations in various populations and exploration of the association with growth outcome through a relevant study design (i.e., a cohort study) could accumulate evidence for more relevant criteria. In addition, the dietary intake of the children was estimated by parents, and the influence of childcare was not considered. Finally, analysis of the association between picky eating behaviors and the adequacy of nutrient intake and growth was stratified by age group; however, the significance of the association was marginal due to the small sample size.

Despite these limitations, the findings from this study should enhance understanding of the association between eating and growth patterns in children. A child’s picky eating behavior has several aspects, although they tend to overlap somewhat. Moreover, different aspects of the behaviors seemed to have a different meaning in terms of a child’s nutritional status. Further study is required to confirm the causality of the observed associations. Investigations of the development of specific picky eating behaviors and long-term outcomes induced by the specific picky eating behaviors are also required. In addition, various attempts to improve the accuracy of classification of the picky eating behaviors are also required.

## 5. Conclusions

This study established concepts for—and measurement of—picky eating behaviors, and assessed the association of picky eating with diet and growth in early childhood. The specific measurement—which consisted of the categories “eating small amounts”, “neophobic behavior”, “refusal of specific food groups”, and “preference for a specific food preparation method”—properly explained the characteristics of various picky eating problems in early childhood. The results of this study suggest that picky eating behaviors—especially eating small amounts of food—are related to insufficient nutrient intake, creating an unfavorable growth pattern. However, the long-term impacts of a diet with limited variety need to be identified.

## Figures and Tables

**Table 1:** Selected characteristics of children aged 1 to 5 years and their caregivers (*n* = 184).

Variables	
***Characteristics of children***	
Age (year), mean ± SD	2.8 ± 1.4
Sex, % boy	48.9
	*n (%)*
Infant feeding practice	
Breastfeeding initiation	177 (96.2)
Exclusive breastfeeding under 3 months of life	166 (90.2)
Exclusive breastfeeding under 6 months of life	93 (50.5)
Introduction of complementary foods ^a^ before 6 months of age	55 (29.9)
Nutrition Plus ^b^ participation	
Yes	72 (39.1)
No	112 (60.9)
Picky eating behavior ^c^	129 (70.1)
Eating small amount	55 (29.9)
Limited variety ^d^	123 (66.9)
Neophobic behavior	60 (32.6)
Refusal of specific food groups	81 (44.0)
Preference for a specific food preparation method	91 (49.5)
	*mean ± SD*
Growth status ( *z*-score)	
Weight for age	0.1 ± 0.8
Height for age	−0.3 ± 1.1
BMI for age	0.3 ± 1.0
***Characteristics of caregivers and the household***	
Age (year), mean ± SD	34.9 ± 3.8
	*n (%)*
Education level of father	
≤High school	18 (9.8)
University	137 (74.5)
Graduate school	29 (15.8)
Education level of mother	
≤High school	29 (15.8)
University	135 (73.4)
Graduate school	20 (10.9)
Household income	
≤$2800	76 (41.3)
$2800 to $3900	58 (31.5)
≥$3900	50 (27.2)

^a^ All foods except breast milk and formula; ^b^ A Nutrition supplemental program for women, infant, and children in Korea; ^c^ Children who had any one of the picky eating constructs: ‘eating small amounts’ and ‘limited variety’; ^d^ Children who had any one of the sub- constructs of limited variety: ‘neophobic behavior’, ‘refusal to eat specific food groups’, and ‘preference for a specific food preparation method’.

**Table 2:** Comparison of nutrient intakes between picky eaters and non-picky eaters according to specific picky eating behaviors (*n* = 184).

	Eating Small Amounts			Neophobic Behavior			Refusal of Specific Food Groups			Preference for Specific Food Preparation Method		
	Yes (*n* = 55)	No (*n* = 129)	*p* ^a^	Yes (*n* = 60)	No (*n* = 124)	*p* ^a^	≥2 (*n* = 81)	0–1 (*n* = 103)	*p* ^a^	≥1 (*n* = 91)	0 (*n* = 93)	*p* ^a^
	mean ± standard deviation											
**Mean daily dietary intake**												
Energy (kcal)	1155 ± 340	1340 ± 348	0.0005	1299 ± 393	1278 ± 336	0.6959	1261 ± 364	1304 ± 348	0.2210	1265 ± 361	1304 ± 350	0.1273
Protein (% Energy)	16 ± 3	16 ± 2	0.8880	16 ± 2	16 ± 2	0.0745	16 ± 2	16 ± 2	0.8004	16 ± 2	16 ± 2	0.4553
Lipid (% Energy)	24 ± 5	24 ± 5	0.4794	24 ± 6	24 ± 5	0.4390	24 ± 5	24 ± 5	0.6578	23 ± 5	24 ± 5	0.8095
Carbohydrate (% Energy)	60 ± 6	61 ± 6	0.5902	60 ± 6	61 ± 6	0.1876	60 ± 6	61 ± 6	0.7830	61 ± 6	60 ± 5	0.9370
Calcium (mg)	416 ± 146	449 ± 217	0.2252	440 ± 209	438 ± 194	0.3546	404 ± 157	466 ± 223	0.0919	411 ± 162	466 ± 227	0.0705
Iron (mg)	8 ± 3	10 ± 4	0.0073	9 ± 3	10 ± 4	0.0938	9 ± 3	10 ± 5	0.0198	9 ± 3	10 ± 5	0.0146
Vit. A (µg RE)	393 ± 205	460 ± 239	0.0370	382 ± 187	468 ± 245	0.0859	386 ± 187	483 ± 252	0.0064	404 ± 199	475 ± 254	0.0231
Thiamin (mg)	0.66 ± 0.24	0.78 ± 0.26	0.0116	0.78 ± 0.29	0.72 ± 0.25	0.5680	0.71 ± 0.27	0.76 ± 0.25	0.0367	0.74 ± 0.26	0.75 ± 0.26	0.1562
Riboflavin (mg)	0.9 ± 0.3	1.0 ± 0.3	0.0072	1.0 ± 0.3	1.0 ± 0.3	0.6564	0.9 ± 0.3	1.0 ± 0.3	0.0444	0.9 ± 0.3	1.0 ± 0.3	0.1868
Niacin (mg)	8 ± 3	9 ± 3	0.0018	9 ± 4	9 ± 3	0.2177	9 ± 3	9 ± 3	0.1640	9 ± 3	9 ± 3	0.1754
Vit. C (mg)	77 ± 55	94 ± 61	0.0380	83 ± 64	92 ± 57	0.1119	82 ± 57	95 ± 61	0.0274	89 ± 67	90 ± 52	0.4510
Total Dietary Fiber (g)	11± 4	13 ± 5	0.0122	12 ± 5	12 ± 4	0.1032	12 ± 5	13 ± 5	0.0110	12 ± 5	13 ± 5	0.0980
**Nutrient density (intake/1000 kcal)**												
Calcium (mg)	371 ± 113	332 ± 111	0.2044	336 ± 102	347 ± 117	0.2556	327 ± 103	357 ± 118	0.3166	328 ± 93	359 ± 127	0.1935
Iron (mg)	7 ± 2	7 ± 3	0.2711	7 ± 1	7 ± 3	0.1159	7 ± 1	8 ± 3	0.0524	7 ± 2	8 ± 3	0.0696
Vit. A (µg RE)	349 ± 172	348 ± 172	0.4670	301 ± 151	371 ± 177	0.1554	315 ± 154	375 ± 181	0.0514	325 ± 146	371 ± 192	0.1247
Thiamin (mg)	0.56 ± 0.12	0.58 ± 0.12	0.3715	0.60 ± 0.13	0.56 ± 0.11	0.1805	0.56 ± 0.13	0.58 ± 0.11	0.0335	0.58 ± 0.11	0.57 ± 0.12	0.6568
Riboflavin (mg)	0.8 ± 0.2	0.8 ± 0.2	0.6383	0.8 ± 0.1	0.8 ± 0.2	0.2690	0.7 ± 0.1	0.8 ± 0.2	0.1702	0.7 ± 0.1	0.8 ± 0.2	0.6353
Niacin (mg)	7 ± 1	7 ± 2	0.0758	7 ± 1	7 ± 1	0.0683	7 ± 1	7 ± 1	0.3064	7 ± 1	7 ± 2	0.7212
Vit. C (mg)	68 ± 35	71 ± 42	0.2579	64 ± 42	72 ± 39	0.1617	65 ± 38	73 ± 42	0.0769	69 ± 42	70 ± 38	0.6559
Total Dietary Fiber (g)	9 ± 3	10 ± 2	0.4781	9 ± 2	10 ± 3	0.0167	9 ± 2	10 ± 3	0.0074	10 ± 2	10 ± 3	0.3655

^a^ *P*-value adjusted for age, sex, and education level of both parents. RE: retinol equivalent.

**Table 3:** Comparison of growth indices between picky eaters and non-picky eaters according to specific picky eating behaviors (*n* = 184).

	Eating Small Amounts			Neophobic Behavior			Refusal of Specific Food Groups			Preference for Specific Food Preparation Method		
Total subjects (*n* = 184)	Yes (*n* = 55)	No (*n* = 129)	*p* ^a^	Yes (*n* = 60)	No (*n* = 124)	*p* ^a^	≥2 (*n* = 81)	0–1 (*n* = 103)	*p* ^a^	≥1 (*n* = 91)	0 (*n* = 93)	*p* ^a^
Growth status (*z*-score)												
Weight-for-age	−0.2 ± 0.9	0.2 ± 0.8	0.0010	0.1 ± 0.9	0.1 ± 0.8	0.9797	0.0 ± 0.9	0.1 ± 0.7	0.4137	0.1 ± 0.8	0.0 ± 0.8	0.2268
Height-for-age	−0.5 ± 1.1	−0.2 ± 1.1	0.0545	−0.5 ± 1.3	−0.2 ± 1.0	0.1057	−0.3 ± 1.1	−0.2 ± 1.1	0.8774	−0.1 ± 1.1	−0.4 ± 1.1	0.0275
BMI-for-age	0.0 ± 1.3	0.4 ± 0.9	0.0278	0.5 ± 1.1	0.2 ± 1.0	0.1329	0.2 ± 0.9	0.3 ± 1.1	0.4653	0.2 ± 0.9	0.3 ± 1.2	0.4831
Children aged 1 to 3 years (*n* = 125)	Yes (*n* = 42)	No (*n* = 83)	*p* ^a^	Yes (*n* = 32)	No (*n* = 93)	*p* ^a^	≥2 (*n* = 52)	0–1 (*n* = 73)	*p* ^a^	≥1 (*n* = 61)	0 (*n* = 64)	*p* ^a^
Growth status (*z*-score)												
Weight-for-age	−0.1 ± 0.9	0.2 ± 0.8	0.0911	0.0 ± 1.0	0.1 ± 0.8	0.8962	0.1 ± 1.0	0.1 ± 0.7	0.6288	0.1 ± 0.9	0.0 ± 0.8	0.5295
Height-for-age	−0.4 ± 1.2	−0.2 ± 1.2	0.3665	−0.7 ± 1.5	−0.1 ± 1.0	0.0657	−0.2 ± 1.2	−0.3 ± 1.2	0.3806	−0.1 ± 1.2	−0.4 ± 1.2	0.1739
BMI-for-age	0.1 ± 1.3	0.3 ± 0.9	0.2833	0.6 ± 1.2	0.1 ± 1.0	0.0575	0.2 ± 0.8	0.2 ± 1.2	0.8383	0.2 ± 0.9	0.3 ± 1.3	0.6564
Children aged 4 to 5 years (*n* = 59)	Yes (*n* = 13)	No (*n* = 46)	*p* ^a^	Yes (*n* = 28)	No (*n* = 31)	*p* ^a^	≥2 (*n* = 29)	0–1 (*n* = 30)	*p* ^a^	≥1 (*n* = 30)	0 (*n* = 29)	*p* ^a^
Growth status (*z*-score)												
Weight-for-age	−0.6 ± 0.7	0.2 ± 0.7	0.0007	0.1 ± 0.8	0.0 ± 0.8	0.9373	−0.2 ± 0.8	0.2 ± 0.8	0.0750	0.1 ± 0.8	0.0 ± 0.8	0.3427
Height-for-age	−0.8 ± 0.7	−0.1 ± 0.8	0.0049	−0.2 ± 0.9	−0.3 ± 0.7	0.8754	−0.5 ± 0.9	−0.1 ± 0.7	0.0450	−0.1 ± 0.8	−0.4 ± 0.8	0.1434
BMI-for-age	−0.2 ± 0.9	0.4 ± 0.9	0.0194	0.3 ± 0.9	0.3 ± 0.9	0.9966	0.2 ± 0.9	0.4 ± 0.9	0.8184	0.2 ± 1.0	0.4 ± 0.9	0.7406

^a^ *P*-value adjusted for age, sex, and education level of both parents. BMI: body mass index.

## Eating Small Amounts

1. In general, at the end of a meal how often has your child eaten the amount you think he/she should eat?

Almost Never				Almost Always
1	2	3	4	5

2. How often do you attempt to persuade your child to eat a food?

Almost Never				Almost Always
1	2	3	4	5

3. Does your child have a good appetite?

Very Bad				Very Good
1	2	3	4	5

## Limited Variety

### Neophobic Behavior

4. How often does your child try new and unfamiliar foods at home?

Almost Never				Almost Always
1	2	3	4	5

5. How willing is your child to enjoy new and unfamiliar food when offered?

Very Unwilling				Very Willing
1	2	3	4	5

### Refusal of Specific Food Groups

6. How often does your child refuse the following foods: beans, vegetables, mushrooms, seaweeds, meat, fish, shrimp, shellfish, eggs, fruits, milk, and yogurt?

**Food**	**Almost** **Never** **1**	**2**	**3**	**4**	**Almost** **Always** **5**	**Not** **Applicable**
Beans	□	□	□	□	□	□
Vegetables	□	□	□	□	□	□
Mushrooms	□	□	□	□	□	□
Seaweeds	□	□	□	□	□	□
Meat	□	□	□	□	□	□
Fish	□	□	□	□	□	□
Shrimp	□	□	□	□	□	□
Shellfish	□	□	□	□	□	□
Eggs	□	□	□	□	□	□
Fruits	□	□	□	□	□	□
Milk	□	□	□	□	□	□
Yogurt	□	□	□	□	□	□

### Preference for a Specific Food Preparation Method

7. Does your child eat any of the following foods only if prepared in a specific way: beans, vegetables, mushrooms, seaweeds, meat, fish, shrimp, shellfish, and eggs?

**Food**	**Almost** **Never** **1**	**2**	**3**	**4**	**Almost** **Always** **5**	**Not** **Applicable**
Beans	□	□	□	□	□	□
Vegetables	□	□	□	□	□	□
Mushrooms	□	□	□	□	□	□
Seaweeds	□	□	□	□	□	□
Meat	□	□	□	□	□	□
Fish	□	□	□	□	□	□
Shrimp	□	□	□	□	□	□
Shellfish	□	□	□	□	□	□
Eggs	□	□	□	□	□	□
